# Over 50 years of trial in Acta Psychiatrica Scandinavica: a survey

**DOI:** 10.1186/1745-6215-10-35

**Published:** 2009-05-27

**Authors:** Hosam E Matar, Muhammad Q Almerie, Muhammad O Al Marhi, Clive E Adams

**Affiliations:** 1 Faculty of Medicine, Damascus University, Khabani Road 2879/FW17, PO Box: 11719, Damascus, Syria; 2 Cochrane Schizophrenia Group, Sir Colin Campbell Building, University of Nottingham Innovation Park, Triumph Road, Nottingham, NG7 2TU, UK

## Abstract

**Background:**

Randomised controlled trials are the gold standard for evaluating mental health care interventions. We assessed the content and quality of trials published in Acta Psychiatrica Scandinavica and Supplementum since 1948.

**Methods:**

All trials were identified manually, quality assessed, data extracted, and sought on Medline.

**Results:**

About 8.6% of all reports in the journal were clinical trials (n = 582) with the peak frequency in the 1980s. Most originate from Europe (80%) and focus on depression (~38%) or schizophrenia (27%). The median sample size is 44. We found only two trials that fully met the criteria of quality reporting RCTs set by CONSORT statements (0.34%) since 1996. Less than 50% of records were possible to identify by a Medline search using broad methodological terms.

**Conclusion:**

Acta is a major source of health trials. The standard of reporting is similar to other journals but better adherence to CONSORT would ensure higher quality of reports and better dissemination.

## Background

Randomised trials are now widely accepted as the "gold standard" for providing evidence on the effectiveness of health care interventions in general [1] as well as mental health care interventions in particular [2]. Random assignment greatly reduces the potential for bias in the allocation of interventions. It ensures that comparison groups are as similar as possible to each other in terms of both known and unknown possible predictors of treatment response [3]. Empirical evidence suggests that non-randomised studies tend to overestimate the effects of healthcare [4]. In addition, the extent and the direction of bias in these studies is often impossible to predict [5].

The use of randomised trials was largely restricted to evaluations of new drug therapies, either against an existing standard agent or against a placebo control. More recently, however, randomised controlled trials are being used to evaluate a much broader range of interventions [6].

Poorly conducted randomised trials, however, can provide biased results [7]. For example, allocation concealment, blinding, intention-to-treat analysis, and loss to follow-up are the critical items in the design and conduct of randomised trials, and an inadequately conceived, conducted and reported randomised trial may overestimate or underestimate true effects of interventions [8,9]. In order to improve design, conduct and reporting of trials CONSORT guidance was drawn up first in 1996 [10] and updated in 2001 [11].

Acta Psychiatrica Scandinavica is one of the leading psychiatric journals with impact factor 3.857 (2006) [12], since the first report of a randomised trial being used in clinical medicine was published in 1948 [13], we aimed to assess the content and quality of trials published Acta Psychiatrica Scandinavica and Acta Psychiatrica Scandinavica Supplementum from 1948 to 2006.

## Methods

All randomised controlled trials or controlled clinical trials were identified by manually searching every page of all relevant volumes of Acta Psychiatrica Scandinavica and Acta Psychiatrica Scandinavica Supplementum from 1948 to Issue 4 of 2006, including Acta Psychiatrica et Neurologica (1948–1950) and Acta Psychiatrica et Neurologica Scandinavica (1951–1961) as Acta predecessors. Working independently, HEM, MQA, and MOA used a standardised data collection sheet to extract information from the papers. Any disagreements were resolved by discussion. We recorded year of publication, type of report, country of origin (address of first author); characteristics of participants including diagnosis, sex, age (adults, elderly or children), setting (hospital or community); variables reporting trial design such as description of randomisation, size, presence of power calculation, consent, duration, parallel or crossover, type of intervention (pharmacological or non-pharmacological) and outcomes.

As allocation concealment is pivotal when describing the likelihood of inclusion of over estimates of effect in trials [14] this was assessed using the Cochrane Collaboration criteria[15]. These criteria consider allocation concealment as 'adequate' when papers report sequentially labelled, sealed, opaque envelopes or central or pharmacy randomisation. By the same criteria, allocation concealment is 'unclear' when papers report randomisation methods used in terms such as "patients were randomised to..." or "treatment was assigned in random order..." Finally, when methods of allocation were explicitly not at random, or by alternation, number of medical record, or date of birth, we categorised these as 'inadequate' randomisation. In addition, each included trial was rated using Jadad scale [16]. This measures a wider range of factors that impact on trial quality. Not only is randomisation rated (adequate = 2, unclear = 1, inadequate = 0), but also double-blindness (adequate = 2, unclear = 1, inadequate = 0) and description of withdrawals (Yes = 1, No = 0). These particular data relevant to quality were chosen because the Jadad rating, in particular, has been validated for mental health trials, and because poor quality scores are associated with over-estimates of effects of treatment [4,8].

We also calculated the proportion of follow up by dividing the number of participants for whom the primary outcome measure was obtained and by the total number randomised. Finally we recorded source of funding of trials (pharmaceutical companies or not) and whether authors were employed by these sources. Electronic records of relevant records on the National Library of Medicine's Medline database, on the PubMed platform, were downloaded into reference manger software (ProCite). We searched for the methodological indexing of all records.

### Statistical analysis

We calculated simple proportions. For trends over time, we grouped data by decade and for this used SPSS (version 13, SPSS Inc., Chicago, IL, USA).

## Results

Acta Psychiatrica Scandinavica and Acta Psychiatrica Scandinavica Supplementum contain 582 reports of clinical trials published between 1953 and 2006. According to PubMed records, this is about 8.6% of all reports published in the Journals since that time. Of these trials, most (423, 73%) were randomised studies but 159 (27%) were 'controlled clinical trials'. In this type of study, randomisation is implied rather than made explicit. Of the 582 trials, most were published as original papers (560, 96%), with only ten (1.7%) being abstracts alone. The remaining 12 (2.3%) were either conference proceedings or letters.

### Reports by Region

**Figure 1:**
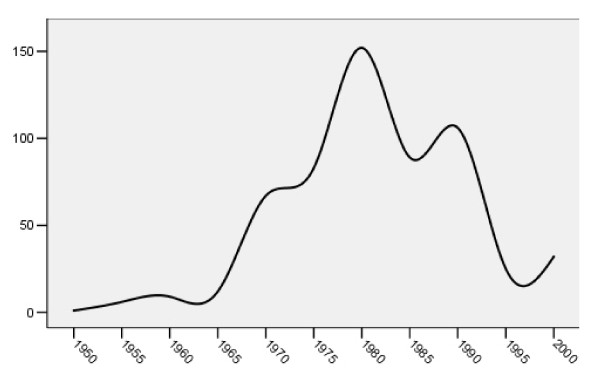
**Number of reports of trials by year of publication**.

The first trial appeared in 1953. Numbers then increased until the 1980's and seemed to peak during that decade (Figure 1). Over 80% (467) of reports of trials were first-authored by someone from Europe, with 138 conducted in Sweden, 69 in Denmark and 67 in the UK (Table 1). Fifty reports (8.6%) originated form the USA. The first paper from Asia was published in 1978 and there has been a steady increase in the published trials originating from there ever since. Thirty percent of studies were carried out in community settings, over 50% in hospitals and 6% in both. For the remainder the setting was unclear.

**Table 1:** Reports by region

Region	Number of reports of trials	%
Europe	467	80.2
North America	63	10.8
Australia	10	1.7
South East Asia	10	1.7
Indian Subcontinent	4	0.7
Middle East	4	0.7
South America	4	0.7
Africa	2	0.3
Not reported	18	3.1
Total	582	100

### Sample size and participants

Data on sample size were available for 577 trials. The median sample size was 44 (IQR 24, 74) with a range [1, 705] and mode of 40. Of the 582 trials, only 11 (1.9%) reported a power calculation. Participants in almost two third of the trials (440 trials, 75%) were both men and women and 546 trials (94%) were conducted on adults. Participants in only seven trials (1.2%) were children and in 29 (5%) were elderly. The diagnosis of trial participants is shown in Table 2. Trials concerned with depression account for nearly 38% of the total and schizophrenia and 'psychoses' studies, another 32%. Reports stated that consent had been gained from participants in 225 trials (39%). The form of the consent was either written or verbal and it may have involved a legal guardian.

**Table 2:** Diagnosis of trial participants

Diagnosis	Number of reports of trials	%
Depression	218	37.5
Schizophrenia	157	27
Healthy Volunteer	48	8.2
Neurotic Disorder	44	7.6
Psychosis	30	5.2
Cognitive Disorder	19	3.3
Sleep Disorder	17	2.9
Addiction Problem	14	2.4
Affective disorder	14	2.4
Organic Disorders	11	1.9
Personality Disorder	10	1.7
Total	582	100

### Trial methods

Most trials (459, 79%) were conducted using a parallel group design, in which participants were allocated to a particular intervention for the entire duration of the study with crossover techniques used in the remainder (123, 21%). Reports stated that allocation to the various interventions was by "randomisation". Over 70% trials did not report the explicit methods by which people were allocated to interventions. Description of allocation concealment was rare (2.4%). We found only two trials that fully met the criteria of quality reporting RCTs set by CONSORT statements (0.34%). Blinding of participants and investigators (double blinding) was employed in 61% of trials but clear descriptions of how this was undertaken and monitored was only reported in 38 (6.5%). Completeness of follow-up was relatively high with over 75% of trials having fewer than 20% participants lost to follow up. Details of the reasons for leaving early were reported in 344 trials (59%). When reporting of allocation, blinding and loss to follow up are compiled into a Jadad score, low scores, 2 or below, indicate poor quality and are associated with over estimation of effects. Overall 353 (61%) of studies in the Journals fell into this category. Proportions of well-reported trials have changed over time (Table 3) but wide confidence intervals indicate that this improvement has not reached statistical significance. The impression of some improvement is paralleled by the increasing impact of Acta [17].

**Table 3:** Jadad score by time and impact factor

		**Jadad score**			
		**0–2**	**3–5 (good quality)**		
	Impact factor	N	N	%	95% CI
1950s	-	7	0	-	-
1960s	-	16	5	23.8	11–45
1970s	-	91	59	39.3	32–47
1980s	-	149	97	39.4	34–46
1990s	1.3–1.7	70	56	44.4	36–53
2000–6	1.7–2.4	20	12	37.5	23–55
Totals		353	229	39.3	35–43

### Interventions and outcomes

Interventions were most commonly pharmaceutical (Table 4) with only small proportions investigating the effects of psychotherapies these studies being particularly rare in depression. Outcomes were measured in many ways but covered the broad categories expected in mental health trials (global state 68%, adverse effects 51%, mental state 20%). Measures or reports of satisfaction with treatment were found in only six (1%) studies, cost electiveness, in four (0.7%) and quality of life, three (0.5%). Most trials were of short duration, with 70 (12%) lasting less than one week and 260 (45%) between one and six weeks. 26% (152) lasted over six weeks to six months, but only 63 (11%) were longer than those periods (37, 6% not reported).

**Table 4:** Interventions used in trials

Types of Interventions	All trials	%	Depression Trials	%	Schizophrenia trials	%
Drug trials	481	82.6	183	83.9	133	84.7
Psychotherapy	40	6.9	3	1.4	12	7.6
ECT trials	28	4.8	19	8.7	4	2.5
Social therapy	6	1.0	0	0	3	1.9
Others	27	4.6	13	6.0	5	3.2
Total	582	100	218	100	157	100

### Source of funding

Trials reported source of funding 183 trials (31.5%) with pharmaceutical companies supporting 82 (45%) and the remaining 101 trials funded by national medical research counsels and academic institutes. Principal investigators in 21/183 trials were directly employed by those providing funding.

### Medline indexing

By searching Medline and Old Medline, we found 566 records for the 582 trials (97.3%). The National Library (NLM), who index for Medline, do not, by policy, index conference abstracts and indexing of letters is somewhat haphazard [18] and it is for this reason that 2.7% of studies in the Actas are not in Medline or Old Medline. Searching the indexing terms by the simple phrase "RANDOM*" identified only 54 records (9.3%). Expanding this to "RANDOM* OR (DOUBLE-BLIND* or (DOUBL* AND BLIND*))" still only identified 273 records (47%). Searching both NLM index terms combined with free text (including abstract and title) by "RANDOM*" identified 237 records (41%) and the "RANDOM* OR (DOUBLE-BLIND* or (DOUBL* AND BLIND*))" phrase 396 records (68%).

## Discussion

This study shows how Acta Psychiatrica Scandinavica and Acta Psychiatrica Scandinavica Supplementum have published a wide range of trials over the last five decades. The proportion of all studies that were RCTs or CCTs (8.6%) being very similar to that in other specialties (8.4%) [19]. Most trials in these European journals originated from Europe. This 'home advantage' has also been demonstrated for other journals [20] but it is encouraging that the proportion of trials from outside Europe in Acta is increasing. We are unclear why the number of trials in the journals seems to be decreasing across time. This may be that studies are being published elsewhere. There is an ever-increasing number of journals vying for authors, Acta's editorial policy or the attractiveness of the journals could have changed over time – for example, by decreasing impact factor. Certainly, other information suggests that there are not fewer mental health trials being published over the same period [20-22].

The content and quality of trials in Acta Psychiatrica Scandinavica and Acta Psychiatrica Scandinavica Supplementum seems similar to those in other major sources [21,22]. Most studies are small (only 1.9% even reported undertaking a power calculation), short (>50% 6 weeks or less) and problematic to apply to everyday care. Moreover, they are so poorly reported that readers have to be concerned as to the validity of their findings. From Table 3 it can be seen that a reasonably high proportion of trials do fall into the 'good quality' category and the impression of improvement (too underpowered to clearly show a statistically significant incline) is paralleled by increasing impact of Acta. This proportion of 'good quality' studies is more than has been seen in preceding similar work [20,21]. However, over 60% of trials continue to have minimal reporting of methods and this is associated with exaggerated estimates of effect [4,8].

We implemented the Jadad scale as it is the only scale that has been developed using established standards for scales and where low scores have been associated with increased effect estimates [23]. However, despite its thorough development and validation, the scale has more focus on the quality of reporting than on methodological quality [24]. As for randomisation, the scale addresses the sequence generation but not concealment of allocation as even adequate concealment of allocation may not prevent against selection bias if the sequence generation is deciphered by the persons enrolling patients [23].

In undertaking a survey of this kind there is a danger of judging work from decades ago by standards of today. However, when this has been applied to other mental health journals findings have been surprising with, for example, the quality of reporting in one major journal declining across time [21]. The CONSORT statement, published over a decade ago, does not seem to have been incorporated into Acta's reporting style.

The indexing of randomised studies could also be improved. Just less than 70% are identified by a broad methodology search in Medline. Even though 123 records did use the terms 'randomised' or 'randomly assigned/allocated' or 'double blind' in the title or abstract of the work, these were not indexed as such by The National Library of Medicine. Medline and Old Medline indexing contains much [understandable] human error. There is, however, a suggestion that 32% of randomised or controlled clinical trials did not mention a methodological term in an obvious way so as to allow easy indexing. It is encouraging that this proportion as steadily decreased across time (95% 1960's, 48% 1970's, 24% 1980's, 16% 1990's), but that 10% of randomised trials since the year 2000 still do not mention key methodological terms in the title or abstract is not good and could be improved by editorial policy. In this way both trialists and editors could be more assured that valuable studies in Acta Psychiatrica Scandinavica and Acta Psychiatrica Scandinavica Supplementum will be identified, used, cited and contribute to impact.

## Conclusion

Acta Psychiatrica Scandinavica and Acta Psychiatrica Scandinavica Supplementum are major sources of mental health trials undertaken in the last half century. The small but increasing proportion of trials from outside of Europe is encouraging, although why the number of trials in the journals is declining is not clear. The standard of reporting is as high, if not higher in these journals compared with their competitors – but could improve with little additional effort. Better adherence to CONSORT would ensure higher quality of reports, better indexing and, as a consequence, wider dissemination.

## Competing interests

The authors declare that they have no competing interests.

## Authors' contributions

HEM, MQA: data extraction, data analysis, writing the manuscript. MOAM: data extraction. CEA: data analysis, writing the manuscript.
